# Effect of cotrimoxazole prophylaxis on malaria occurrence in HIV-infected patients on antiretroviral therapy in sub-Saharan Africa*

**DOI:** 10.1111/tmi.12463

**Published:** 2015-02-06

**Authors:** R Kasirye, K Baisley, P Munderi, H Grosskurth

**Affiliations:** 1 London School of Hygiene and Tropical MedicineLondon, UK; 2 MRC/UVRI Uganda Research Unit on AIDSEntebbe, Uganda

**Keywords:** malaria, cotrimoxazole, HIV, antiretroviral therapy

## Abstract

**Objective:**

To systematically review the evidence on the effect of cotrimoxazole (CTX) on malaria in HIV-positive individuals on antiretroviral therapy (ART).

**Methods:**

Web of Science, PubMed and MEDLINE, EMBASE, Global Health and Cochrane Library databases were searched using terms for malaria, HIV and CTX. Studies meeting the inclusion criteria were reviewed and assessed for bias and confounding.

**Results:**

Six studies (in Uganda, Kenya, Malawi, Zambia and Zimbabwe) had relevant data on the effect of CTX on malaria in patients on ART: four were observational cohort studies (OCS) and two were randomised controlled trials (RCTs); two were in children and one in women only. Samples sizes ranged from 265 to 2200 patients. Four studies compared patients on ART and CTX with patients on ART alone; 2 (RCTs) found a significant increase in smear-positive malaria on ART alone: (IRR 32.5 CI = 8.6–275.0 and HR 2.2 CI = 1.5–3.3) and 2 (OCS) reported fewer parasitaemia episodes on CTX and ART (OR 0.85 CI = 0.65–1.11 and 3.6% *vs*. 2.4% of samples *P* = 0.14). One OCS found a 76% (95% CI = 63–84%) *vs*. 83% (95% CI = 74–89%) reduction in malaria incidence in children on CTX and ART *vs*. on CTX only, when both were compared with HIV-negative children. The other reported a 64% reduction in malaria incidence after adding ART to CTX (RR = 0.36, 95% CI = 0.18–0.74). The 2 RCTs were unblinded. Only one study reported adherence to CTX and ART, and only two controlled for baseline CD4 count.

**Conclusion:**

Few studies have investigated the effect of CTX on malaria in patients on ART. Their findings suggest that CTX is protective against malaria even among patients on ART.

**Objectif:**

Analyser systématiquement les données sur l'effet du cotrimoxazole (CTX) sur le paludisme chez les personnes VIH positives sous traitement antirétroviral (ART).

**Méthodes:**

Web of Science, PubMed et Medline, Embase, Global Health et les bases de données de Cochrane Library ont été recherchés en utilisant des termes pour paludisme, VIH et CTX. Les études répondant aux critères d'inclusion ont été examinées et évaluées pour les biais et les facteurs confusionnels.

**Résultats:**

Six études (en Ouganda, Kenya, Malawi, Zambie et Zimbabwe) avaient des données pertinentes sur l'effet du CTX sur le paludisme chez les patients sous ART; 4 étaient des études de cohortes d'observation (ECO) et deux des essais contrôlés randomisés (ECR). Deux études étaient sur des enfants et une sur des femmes seulement. La taille des échantillons variait de 265 à 2200 patients. Quatre études ont comparé des patients sous ARV et CTX avec des patients sous ART seul. Deux ECR ont constaté une augmentation significative de cas de paludisme à frottis positifs chez les individus sous ART seul: (IRR= 32,5; IC: 8,6 à 275,0 et HR = 1,5; IC: 3,3 – 2,21) et deux ECO ont rapporté moins d’épisodes de parasitémie chez les individus sous CTX et ART (OR 0,85; IC: 0,65 à 1,11 et de 3,6% contre 2,4% des échantillons, P = 0,14). Une ECO a trouvé une réduction de 76% (IC95%: 63-84%) contre 83% (IC 95% = 74-89%) de l'incidence du paludisme chez les enfants sous CTX et ART que chez ceux sous CTX seul, lorsque les deux groupes étaient comparés à des enfants VIH négatifs. L'autre étude fait état d'une réduction de 64% de l'incidence du paludisme après l'ajout de l’ART au CTX (RR = 0,36, IC95%: 0,18 à 0,74). Les deux ECR n’étaient pas réalisées en aveugle. Seule une étude a rapporté l'adhésion au CTX et à l’ART, et seule 2 études ont effectué des ajustements pour la numération des CD4 initiale.

**Conclusion:**

Peu d’études ont évalué l'effet du CTX sur le paludisme chez les patients sous ART. Leurs résultats suggèrent que le CTX est protecteur contre le paludisme, même chez les patients sous ARV.

**Objetivo:**

Revisar de forma sistemática la evidencia del efecto del cotrimoxazol (CTX) sobre la malaria en individuos VIH positivos recibiendo terapia antirretroviral (TAR).

**Métodos:**

Se realizaron búsquedas en las bases de datos de Web of Science, Pubmed y Medline, Embase, Global Health y Librería de Cochrane utilizando los términos malaria, VIH y CTX. Los estudios que cumplían con los criterios de inclusión fueron revisados y evaluados en busca de sesgo y variables de confusión.

**Resultados:**

Seis estudios (en Uganda, Kenia, Malawi, Zambia y Zimbabue) tenían datos relevantes sobre el efecto del CTX en la presencia de malaria en pacientes recibiendo TAR: 4 eran estudios de cohortes y observacionales (ECO) y 2 eran ensayos aleatorizados y controlados (EACs); 2 eran en niños y 1 en mujeres solamente. Los tamaños muestrales estaban entre 265 y 2200 pacientes. Cuatro estudios comparaban pacientes recibiendo TAR y CTX con pacientes recibiendo TAR solamente; 2 (EAC) encontraban un aumento significativo en malaria con frotis positivo entre pacientes recibiendo solamente TAR: (IRR 32.5 IC=8.6-275.0 y HR 2.21 IC=1.5-3.3) y 2 (OCS) reportaron un menor número de episodios de parasitemia en pacientes recibiendo CTX y TAR (OR 0.85 IC=0.65–1.11 y 3.6% versus 2.4% de las muestras P=0.14). Un ECO encontró un 76% (IC 95% = 63–84%) versus 83% (IC 95% =74–89%) en la reducción de incidencia de malaria en niños recibiendo CTX y TAR versus recibiendo solamente CTX, cuando ambos eran comparados con niños VIH negativos. El otro reportaba un reducción del 64% en la incidencia de malaria después de añadir TAR al CTX (RR=0.36, IC 95% =0.18–0.74). Los 2 EACs eran abiertos (no ciegos). Solo1 estudio reportaba adherencia al CTX y TAR, y solo 2 controlaban el conteo de CD4 basal.

**Conclusión:**

Pocos estudios han investigado el efecto del CTX sobre la malaria en pacientes recibiendo TAR. Sus hallazgos sugieren que el CTX protege frente a la malaria incluso en pacientes recibiendo TAR.

## Introduction

Malaria and HIV infection are important global health problems, and these diseases have a wide geographical overlap resulting in frequent co-infection 1–4. HIV infection is associated with deterioration of the patient's immune system and an increased incidence of opportunistic infections (OI) and of malaria 5–8. Among HIV-infected patients, the use of daily prophylaxis with cotrimoxazole (CTX) reduces mortality and morbidity from OI 9–15, and it reduces malaria incidence in HIV-infected patients before 12,16,17 and after starting antiretroviral therapy (ART)^18^,^19^, and in children exposed to HIV infection^20^.

WHO 21 recommends ART for HIV-infected adults and adolescents with a CD4 count <500 cells/μl or if a person has TB, is pregnant, is breastfeeding, HBV co-infected with severe liver disease or in a sero-discordant partnership. WHO also recommends CTX prophylaxis for anybody with a CD4 count <350 cells/μl, or clinical stage 3 and 4 disease and irrespective of CD4 count or clinical stage in areas of high malaria prevalence and/or severe bacterial infections 22. This policy aims to reduce OI and all-cause mortality. The possible preventive effect on malaria was not originally part of the rationale for CTX prophylaxis. Indeed, fears about the possible development of resistance against antimalarial drugs owing to wide-spread CTX use were expressed by some authors 23–25.

CTX use increases patients' pill burden and cost of care and is associated with haematological toxicity and hypersensitivity skin reactions. However, adverse reactions to CTX are rare (<2 per 100 person-years of CTX use) and mainly mucocutaneous in nature; they resolve with drug discontinuation 26–28. With patients on ART being able to regain near normal immune function, some researchers recommend stopping CTX once patients are stable on ART 29,30. However, these recommendations are based on studies in industrialised countries which have fewer malaria and bacterial infections than sub-Saharan Africa (SSA). So although HIV-infected patients on ART in SSA may not need CTX for prevention of OI, they might still benefit from its antimalarial effect 31. A Guideline Development Group on Cotrimoxazole prophylaxis was convened by WHO in 2013. This group recommended that in settings with high malaria prevalence and/or severe bacterial infections, even among patients that are stable on ART, CTX should be continued 22.

Our objectives were (i) to systematically review publications on the effect of CTX on malaria in HIV-infected patients on ART in order to assist policymakers in SSA in taking informed decisions within the context of the epidemiological situation in their area and (ii) to provide background information for an ongoing controlled trial of malaria incidence among HIV-infected patients on ART with and without CTX co-medication.

## Methods

### Search strategy

The following databases were searched for publications to 14 April 2014: EMBASE, PubMed and MEDLINE, Web of Science, Global Health and the Cochrane Library. The search used terms for malaria, HIV and CTX, without a term for ART, to reduce the chance of missing relevant papers.

A combination of the following MESH terms and free text was used:

- *malaria, malaria incidence, malaria prevalence, malaria severity, malaria outcomes, malaria treatment, Plasmodium* and *parasitaemia* for malaria.
- *cotrimoxazole, trimethoprim/sulfamethoxazole, septrin* and *bactrim* for cotrimoxazole.
- *HIV, human immunodeficiency virus, acquired immunodeficiency syndrome, AIDS* and *immune suppression* for HIV infection.

An example of the search strategy as used in MEDLINE

- malaria OR malaria adj3 (occurrence OR incidence OR prevalence OR treatment OR parasit?emia OR outcomes OR sever*) OR Plasmodium.
- HIV OR human immunodeficiency virus OR acquired immunodeficiency syndrome OR AIDS OR Immune suppres*.
- 1 AND 2.
- cotrimoxazole OR trimethoprim sulfamethoxazole OR septrin OR bactrim.
- 3 AND 4.

The search results were exported to Endnote reference management software (Thomson Reuters, version ×7) and duplicates were removed. All titles and abstracts were screened independently by two authors (RK and KB); inconsistencies were discussed and consensus on potential eligibility reached. Abstracts were checked for studies reporting on a combination of malaria, HIV, cotrimoxazole and antiretroviral therapy. Review articles were excluded but their reference lists were checked for possible additional relevant papers. Reference lists from papers identified from the systematic search were also checked. Full text copies of potentially relevant papers were then obtained. Data requests were sent to authors of studies for which relevant information might have been collected but not reported in their publications. Guidelines on preferred reporting items for systematic reviews and meta-analyses (PRIMSA) were used.

### Included studies

We included studies containing original data on the effect of CTX on malaria in HIV-infected patients on ART. No restrictions on area of the world, participant age, language or the date of publication were used.

### Data extraction

Data from papers identified by the search were independently extracted by two authors (RK and KB) using a standard form to collect the following information: first author's name, year of publication, type of study, study population, study aim, sample size, follow-up time, study results, how malaria diagnosis was made, number and severity of malaria episodes and the association between malaria and CTX.

### Bias

Assessment of bias and confounding within studies and quality of papers was based on PRISMA guidelines^32^ and the Newcastle-Ottawa quality assessment of studies scale^33^. Studies were assessed on selection bias, adherence to CTX, objectivity of malaria diagnosis, cohort retention, follow-up duration (for assessment of seasonal variation), adjustment for confounding and outcome reporting. A formal meta-analysis was not performed owing to the diversity in study methodologies, comparison groups and populations.

## Results

**Figure 1:**
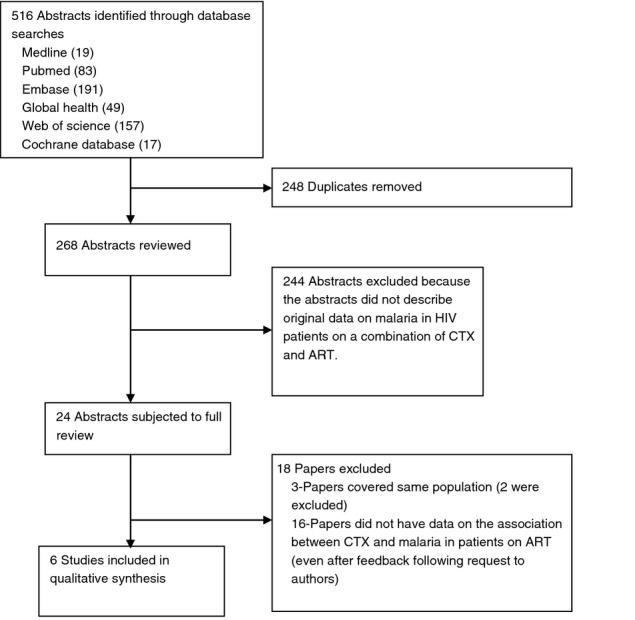
Results of the systematic search.

A total of 516 abstracts were retrieved, of which 492 were removed because they were duplicates, were not relevant or did not meet the inclusion criteria (Figure 1). Full text was reviewed for 24 abstracts; only 6 had data on the effect of CTX on malaria in HIV-infected patients on ART and were retained for the final qualitative synthesis. 16 potentially eligible studies did not report on the association of CTX and malaria in patients on ART; authors of 14 papers were contacted where it was felt that relevant data might have been collected but not reported. However, none was able to provide information relevant to this review so the studies were not included.

All six studies included in the final synthesis were conducted in SSA; no relevant studies were found from malarious areas in other continents. Of the six studies, two were randomised controlled trials and four were observational cohort studies; two studies were conducted among children only and four among adults (one in women only) (Table1). Four studies were conducted in Uganda only (or the analysis of malaria was restricted to Ugandan sites); the other two were multisite studies conducted in Kenya, Malawi, Uganda, Zambia or Zimbabwe. The diagnosis of malaria was based on a patient having history of fever and a positive blood slide or rapid diagnostic test (RDT) (4 studies); clinical features alone or clinical features and a positive blood slide (1 study); and a positive blood slide or positive RDT, or detection of *Plasmodium falciparum* histidine rich protein 2 in plasma (1 study). Study samples sizes ranged from 265 to 2200 patients. Median length of follow-up was not specified for one study and was between 4 months and 4.9 years for the others. A summary of studies in the final synthesis is shown in Table1.

**Table 1:** Summary of studies on the effect of CTX on malaria in HIV-infected patients on ART

Author/year	Type of study	Study population	Main study aim	Number of participants (median follow-up)	Main study or non-malarial outcome. Ratio (95% CI)	Malaria diagnosis (number of episodes).	Malaria comparison by CTX/ART	Association between malaria and CTX. Ratio (95% CI)
Bwakura-Dangarembizi 2014^34^	RCT	Children on ART (Uganda and Zimbabwe)	Assess the effect of stopping *vs*. continuing CTX in children on ART	758 (2.1 years)	Stopping CTX associated with higher rates of hospitalisation or death. HR 1.64 (1.14–2.37 *P* = 0.007)	Positive smear or RDT (169)	ART only *vs*. CXT/ART	HR 2.21 (1.50–3.25; *P* < 0.001) Median parasite density (221 *vs*. 153) Hospitalisation for malaria (49 *vs*. 21)
Campbell/2012^35^	RCT	Adults on ART (Uganda)	Assess effect of stopping CTX on malaria and diarrhoeal incidence	836 (4 months*)	Stopping CTX associated with higher incidence of diarrhoea IRR 1.8 (1.3–2.4, *P* < 0.001)	Smear positive fever (57)	ART only *vs*. CXT/ART	IRR 32.5 (8.6–275.0; *P* < 0.001) Parasite density >1250 parasites/μl (70% *vs*. 100%)
Gasasira/2010^12^	Cohort	HIV-infected and uninfected children (Uganda)	Assess protective efficacy of CTX on malaria and prevalence of CTX resistance mutations in *P*. falciparum	517 HIV-uninfected (2.1 years) and 292 HIV-infected (2.4 years)	Prevalence of DHFR and DHPS mutations was >90%. Efficacy of CTX on malaria (HIV infected *vs*. uninfected) was 80% (72–85%)	Smear positive fever (576 total, 65 in HIV positive)	Efficacy† (CTX with ART *vs*. HIV negative; CTX only *vs*. HIV negative)	CTX and ART: efficacy = 76% (63–84%) CTX only: efficacy = 83% (74–89%)
Mermin/2006^19^	Cohort	HIV-infected adults (Uganda)	Assess the effect of ART on malaria and additive effects of CTX, ART and ITNs. Had 4 phases; *one*-no intervention (NI), *two***-**CTX, *three-*CTX and ART, *four-*CTX, ART and ITNs	Phase; *one* 466 (154 days), *two* 399 (532 days), *three* 1035 (126 days), *four* 989 (560 days)	Adjusted IRR Cumulative (phase *one* as reference) *CTX vs. NI* 0.24 (0.12–0.17) *P* < 0.001 *CTX/ART/ITNs vs. NI* 0.05 (0.03–0.08) *P* < 0.001 Additive effect (the previous phase as the reference) *CTX vs. NI* 0.24 (0.15–0.38) *P* < 0.001 *CTX/ART/ITNs vs. CTX/ART* 0.58 (0.31–1.11) *P* = 0.1	Smear positive fever smear. (166)	Cumulative CTX/ART *vs*. NI Additive CTX/ART *vs*. CTX	Cumulative 0.08 (0.04–0.17) *P* < 0.001 Additive effect 0.36 (0.18–0.74) *P* = 0.006 Similar rates observed when malaria defined as parasitaemia >1250 μl
Skinner Adams/2012^36^	Cohort	HIV-infected women in OCTANE trial‡ (Kenya, Uganda, Malawi, Zambia)	Assess effect of LPV/R compared to nevirapine-based ART on malaria	265§	Samples positive for malaria in subjects receiving LPV/R compared to those receiving NVP-based ART (2.8% vs. 1.8%, *P* = 0.13)	Positive smear, RDT or malaria antigen in plasma (104)	ART *vs*. CTX and ART only	Number of positive samples; Analysing one episode per subject 2.9% *vs*.. 2.2% *P* = 0.42 Allowing multiple episodes per subject 3.6% *vs*. 2.4% *P* = 0.14
Walker/2010^18^	Cohort	HIV-infected adults in the DART trial¶ (Ugandan sites)	Assess effect of CTX on survival, WHO stage, malaria, CD4, BMI and haematological indices after initiating ART	2200 (4.9 years)**	Being on CTX *vs*. being off CTX; Mortality (0.65, 0.50–0.85)	2362 events (Clinically 1243, microscopically 1119)	CTX/ART *vs*. ART	Clinical and laboratory diagnosis OR = 0.74 (0.63–0.88) *P* < 0.001. When restricted to parasite positive diagnoses OR = 0.85 (0.65–1.11) *P* = 0.23

RCT, Randomised controlled trial; IRR, incidence rate ratio; DHFR, dihydrofolate reductase; DHPS, dihydropteroate synthetase; BMI, body mass index; OR, odds ratio; LPV/R, lopinavir/ritonavir; RDT, rapid diagnostic test; DART, Development of Antiretroviral Therapy; ITNs, insecticide-treated bed nets.

* Total fup time.

† Protective efficacy (1-IRR).

‡ Octane (A5208) trial sites with malaria; Kericho Kenya, Lilongwe Malawi, Kampala Uganda, Lusaka Zambia.

§ Prevalence in samples, no follow-up time.

¶ Development of Antiretroviral Therapy trial sites with malaria; Kampala and Entebbe, Uganda.

** Median fup is across all sites (Uganda and Zimbabwe).

## Summary of study objectives and populations

Bwakura-Dangarembizi *et al*. 34 conducted a randomised, open-label, controlled trial in Uganda and Zimbabwe to assess the effect of stopping *vs*. continuing CTX prophylaxis in HIV-infected children and adolescents on long term ART. The trial enrolled 760 participants with median age of 7.9 years; at enrolment, the median time on ART was 2.1 years and median CD4 T-cell percentage was 33% (nadir-13%).

Campbell *et al*. 35 conducted a cluster randomised (by household) controlled trial in Uganda to investigate the effect of CTX discontinuation on the incidence of malaria and diarrhoea among HIV-infected adults on ART with CD4 > 200 cells/μl. The trial enrolled 836 participants; at enrolment, the median time on ART was 3.7 years, median CD4 was 489 (nadir = 129) cells/μl, and 94% had a viral load <100 copies/μl.

Gasasira *et al*. 12 assessed the protective efficacy of CTX on the incidence of falciparum malaria and on the prevalence of *Plasmodium falciparum* mutations conferring antifolate resistance among children treated for malaria in Uganda, comparing HIV-infected children on daily CTX both on and off ART, and HIV-uninfected children not taking CTX. The study enrolled 899 children (300 HIV infected) with a median age of 7.4 (HIV-uninfected) and 5.7 years (HIV infected) at enrolment. The median CD4 T-cell percentage was 23% (HIV-infected). HIV-infected children contributed 665 person-years of follow-up, of whom 275 were on ART (292 participants).

Mermin *et al*. 19 assessed the effect of ART on malaria and the additive effects of CTX, ART and insecticide-treated bed nets (ITNs) in HIV-infected adults attending clinics of The AIDS Support Organization (TASO) at two sites in Uganda. Study participants had sequential exposure to the intervention divided into four phases:

- Phase 1 – no intervention (466 participants; median CD4 at enrolment = 75 cells/μl)
- Phase 2 – participants started on CTX prophylaxis (399 participants; median CD4 at enrolment = 77 cells/μl)
- Phase 3 – participants continued CTX and started on ART (1035 participants; median CD4 at enrolment = 124 cells/μl)
- Phase 4 – participants continued CTX and ART, and ITNs were provided (985 participants; median CD4 at enrolment = 175 cells/μl)

Skinner *et al*. 36 assessed the effect of a protease inhibitor (PI)-based ART regimen on malaria compared with a nevirapine-based regimen, stratified by CTX use. Patients were part of the Optimal Combination Therapy After Nevirapine Exposure study (OCTANE) 37, a multicentre trial comparing non-nucleoside reverse transcriptase inhibitor (NNRTI) and PI-based regimens for HIV-infected women with a history of nevirapine prophylaxis to prevent mother-to-child HIV transmission. The Skinner *et al*. study evaluated 265 women from the OCTANE trial who at baseline had a median age of 37 years, median CD4 of 121 cells/μl and HIV RNA of 5.2 log10 copies/ml.

Walker *et al*. 18 assessed the effect of CTX on survival, WHO stage, malaria, CD4 count, body mass index (BMI) and haematological indices in HIV-infected patients with CD4 count <200 cells/μl initiating ART in Uganda and Zimbabwe in the Development of Antiretroviral Therapy (DART) trial for Africa which compared ART monitoring strategies in resource limited settings. The trial enrolled 3179 participants; the analysis of malaria incidence was restricted to 2222 participants in Uganda. At enrolment into the DART trial, median CD4 count was 83 cells/μl.

## Effect of CTX on malaria in patients on ART

Two of the six studies evaluated the effect of CTX on malaria in HIV-positive participants on ART as their main study objective. The other four studies had different objectives, but the data allowed assessment of the effect of CTX on malaria.

The six studies used different comparison groups: CTX and ART *vs*. ART alone (4 studies); CTX and ART *vs*. HIV negative (1 study); CTX only *vs*. HIV negative (1 study); CTX only *vs*. HIV positive not on treatment (1 study); CTX and ART *vs*. HIV positive not on treatment (1 study); CTX, ART and ITNs *vs*. HIV positive not on treatment (1 study).

All four studies that examined the occurrence of malaria in HIV-positive persons on CTX and ART compared with those on ART alone found a beneficial effect of CTX. Bwakura-Dangarembizi *et al*. 34 found that children and adolescents who discontinued CTX had a higher incidence of malaria (HR 2.21, 95% CI = 1.50–3.25 *P* < 0.001) compared to those who continued CTX prophylaxis. Campbell *et al*. 35 also found strong evidence of a higher malaria incidence in patients on ART who discontinued CTX compared to those who continued CTX prophylaxis (IRR 33; 95% CI = 9–275, *P* < 0.001). Walker *et al*. 18 found strong evidence of a reduction in the number of clinical malaria episodes among patients on ART and CTX compared to those on ART alone (OR = 0.74, 95% CI = 0.63–0.88, *P* < 0.001); however, the reduction in the risk of parasitaemia was not statistically significant (OR 0.85; 95% CI = 0.65–1.11, *P* = 0.23). Skinner *et al*. 36 found weak evidence of decrease in detectable parasitaemia in patients who were on CTX and ART compared to those who were on ART alone (3.6% *vs*. 2.4%; *P* = 0.14).

Gasasira *et al*. 12 found a 76% (95% CI = 63–84%) reduction in malaria incidence in children on CTX and ART, and a similar 83% (95% CI = 74–89%) reduction in children on CTX only, when both were compared with HIV-negative children not on CTX.

Mermin *et al*. 19 found that CTX alone was associated with 76% reduction in malaria incidence (RR = 0.24, 95% CI = 0.15–0.38; *P* < 0.001), and CTX and ART reduced malaria incidence by 92% (RR = 0.08, 95% CI = 0.04–0.17; *P* < 0.001), when compared with HIV-positive participants not on CTX or ART. In their sequential comparisons of the additive effects of the interventions in each phase of the study, they found that adding ART to CTX was associated with a 64% (RR = 0.36, 95% CI = 0.18–0.74; *P* = 0.006) reduction in malaria incidence compared to CTX alone.

Four studies reported parasite density. Bwakura-Dangarembizi *et al*. 34 found parasite density to be higher in patients on ART who stopped CTX compared with those who continued CTX (median parasite density per 200 white cells = 221/μl *vs*. 153/μl, *P* = 0.004). Campbell *et al*. 35 found that 70% of the 55 malaria episodes in patients on ART who stopped CTX had parasite densities >1250/μl compared with 100% of the two episodes in patients who continued CTX. Gasasira *et al*. 12 found that geometric mean parasite density was lower for HIV-infected children who were also on CTX (6462/μl) compared to HIV-uninfected children (11 270/μl), although the difference was not statistically significant (*P* = 0.40). Mermin *et al*. 19 evaluated the cumulative and additive effects of CTX, ART and ITNs on malaria parasitaemia >1250/μl; their conclusions were similar to those when malaria was defined as fever with a positive blood slide (as described above).

### Risk of bias and confounding

All studies reviewed were of good quality but subject to sources of bias (Table2). Three studies reported adherence to CTX (Bwakura-Dangarembizi, Campbell and Gasasira *et al*.), and 3 (Bwakura. Mermin and Walker *et al*.) reported adherence to ART. Only the Bwakura *et al*. study reported both CTX and ART adherence.

**Table 2:** Risk of bias and confounding within studies

Criterion	Study Author, year					
Assessment of bias	Bwakura-Dangarembizi 2014 34	Campbell 2012 35	Gasasira 2010 12	Mermin 2006 19	Skinner 2012 36	Walker 2010 18
Ascertainment of exposure (adherence to CTX and ART)	No difference between groups in adherence to ART. Self-report: 6% had missed CTX doses during the previous 4 weeks	ART not reported, CTX adherence in cont. CTX group not mentioned	Median level of CTX adherence in HIV-infected population was 100%. ART adherence not mentioned	95% ART adherence, CTX not reported	Not reported. From the main trial: trial 1, 81% in LPV/r AND 83% in NVP arm took 95% of expected doses. In trial 2, adherence to ART at each visit = 84–92% No information about CTX adherence	ART adherence - no missed doses reported at 83% of visits in those on CTX and at 78% of visits in those not on CTX in first 12 wks; 93% and 87% of visits in weeks 12–72 93% and 91% of visits >72 weeks on ART. CTX not reported
Ascertainment of malaria diagnosis	Positive slide/RDT	Fever in past 7 days and positive slide	Fever in past 24 h and positive slide	Fever in previous 2 days and positive slide	Positive slide, RDT or antigen in plasma	Clinically and or microscopically
Randomised by CTX in patients on ART	Yes	Yes	No	No	No	No
Study groups comparable at baseline	Yes	No (mean CD4 higher in stop CTX arm)	No	No	N/A	No
Participants/investigators blinded to CTX use	No	No-only laboratory technicians were blinded	No	No	No	No
Loss to follow-up	7 in stop CTX arm (2%) and 2 in CTX arm (0.5%)	0% (short fup)	13% in HIV neg: 6% in HIV positive (not given by ART status)	<10% all three phases	N/A (data analysed on a cross sectional basis) Main trial: 2.5% in trial 1 and 6% in trial 2	6%
More than 1-year follow-up (seasonal variation)	Yes	No	Yes	Yes, overall and phase 2 and 4 but not in phase 1 and 3	N/A	Yes
Control for potential confounders						
Baseline CD4 cell count	Study design	No*	No	Analysis	No	Analysis
ITN use	No†	Study design	No	Study design	No	No
Age	Study design	Study design	Analysis	Analysis	No	Analysis
Sex (Gender)	Study design	Study design	No	Analysis	Study design	Analysis
Socio-economic status	Study design	Study design	No	No	No	No
Other	Stratification by randomisation factors	Clustering by household not adjusted for	Breast feeding not controlled for	Season, adjust for in analysis	Multivariate analysis not carried out	Length of time on ART. Used MSM to control for time dependent confounding

N/A, Not applicable; No, confounder not controlled for; RDT, rapid diagnostic test.

* Selection criterion in this study was applied after randomisation giving a difference in CD4 count as baseline.

† ITN use reported to be higher in patients stopping CTX (*P* = 0.02). MSM, marginal structural models.

Campbell *et al*. and Bwakura-Dangarembizi *et al*. were the only studies randomised by CTX use; however, these studies were not blinded. Most of the studies used clinical and/or laboratory based methods to diagnose malaria; Walker *et al*. also used clinical diagnosis alone.

Multivariate analysis to control for potential confounders was not used by Skinner *et al*. due to the small number of malaria cases. Only the Mermin, Bwakura-Dangarembizi and Walker studies controlled for the potential confounding effect of CD4 count at baseline. No study explored the potential confounding effect of socio-economic status. The Walker study was the only study to control for potential time-dependent confounders such as current CD4 count, haemoglobin and BMI levels.

## Discussion

Most studies in this review were conducted after 2005, when most developing countries had started to roll out ART to patients, of whom most were on CTX. The search was performed using terms for malaria, HIV and CTX, without using the term for ART, to reduce the chance of missing relevant papers. However, only six studies were identified with data on the effect of CTX on malaria in patients on ART.

Four of the six studies compared malaria occurrence in patients on ART alone with that in patients on ART and CTX, and all found a higher incidence of malaria in patients on ART alone. This is expected given the antimalarial properties of CTX^38^, even in areas where malaria parasites have antifolate resistance^12^. The strongest evidence for this beneficial effect was observed in the Campbell (IRR = 32.5) and Bwakura-Dangarembizi (HR = 2.21) studies. The latter study was conducted in children and adolescents and the former in adults. With better immune memory to malaria in adults^39^, a smaller difference in malaria incidence between those stopping CTX and those continuing CTX might be expected in adults than in children, but the reverse was observed. The Campbell study was stopped after just 4 months because of increased malaria incidence in the discontinuation arm. As the authors point out, it is not clear whether the increase in malaria after stopping CTX may have been only temporary. It is possible that the larger beneficial effect of CTX on malaria in adults in this study, compared with that in children and adolescents in the Bwakura-Dangarembizi study, is because of the shorter follow-up time (4 months *vs*. 2.1 years, respectively).

The other two studies showed strong evidence of a decrease in episodes of clinical malaria (Walker *et al*. OR = 0.74, 95% CI = 0.63–0.88; *P* < 0.001), and weak evidence of a decrease in malaria prevalence (Skinner et al. 3.6% *vs*. 2.4%, *P* = 0.14), in patients on ART and CTX compared to patients on ART alone. One study showed that HIV-infected children on CTX had 80% (95% CI; 72–85%) lower malaria incidence than HIV-negative children not on CTX.

Only the Bwakura-Dangarembizi and Campbell studies were originally designed to look at the effect of CTX on malaria in patients on ART. This may explain why most studies did not report adherence to CTX and/or ART and did not attempt to address potential bias and confounding related to malaria. Both the Bwakura-Dangarembizi and Campbell studies were randomised by CTX use, but investigators and participants were not blinded. These trials showed that patients stopping CTX prophylaxis had a higher risk of malaria than those who continue CTX. The Bwakura-Dangarembizi study reported more hospitalisations in children stopping CTX than in those who continued. The Campbell study showed no significant difference in adult hospitalisation rates or mortality between the trial arms, and none of the four deaths recorded was related to malaria. Malaria in this study was uncomplicated and the clinical relevance of this malaria is therefore not clear.

Results of a trial ‘CTX Prophylaxis Discontinuation Among ART-Treated Adults: A Randomized Non-Inferiority Trial’ were presented at the Conference on Retroviruses and Opportunistic Infections in March 2014^40^. These results were not included in this synthesis because some data relevant to the review were not provided and the results are yet to be published in a peer-reviewed journal. This trial was an open-label randomised controlled trial comparing stopping *vs*. continuing CTX prophylaxis among 500 HIV-infected adults in western Kenya on ART >18 months and followed for a year. The authors reported that patients stopping CTX had a 33.2 (95% CI = 4.5–241.0; *P* = 0.001) times higher malaria incidence than those who continued CTX. They also found that combined morbidity and mortality were greater in the group stopping CTX (IRR 2.27; 95% CI = 1.52–3.38, *P* < 0.001) but that this result was driven by malaria morbidity.

Publication of the full results of this trial is awaited but like the Campbell study, this trial was not blinded and therefore susceptible to reporting bias. However, the results suggest that the higher incidence of malaria seen in patients stopping CTX in the Campbell study may be maintained even when patients are followed for longer.

It is clear from this review that patients who are stable on ART and stop taking CTX experience malaria episodes more frequently but a number of questions remain unanswered:

- Given that none of the studies was blinded, how much does reporting bias contribute to the observed increased risk of malaria in patients who stop CTX compared to that in those who continue?
- What is the clinical significance of malaria occurring in HIV-infected adults on ART who do not take CTX prophylaxis?
- Do patients who stop CTX have a higher incidence of malaria than would be observed in HIV-uninfected people?
- How does CTX compare with other antimalaria prophylactic drugs such as chloroquine (which may imply a lower pill burden)?
- Does the background ecological exposure to malaria have an effect on the relationship between malaria and CTX?
- Is CTX prophylaxis still beneficial in patients recently diagnosed with HIV who start ART early, that is with high CD4 counts, for example 500 cells/μl?

Two ongoing randomised controlled trials, one in Uganda (ISRCTN44723643) and one in Malawi (NCT01650558), will help answer some of these questions. These studies investigate the effect of CTX on malaria incidence in HIV-infected patients on ART. The Ugandan trial is double-blind and placebo-controlled, and compares continued CTX prophylaxis with stopping CTX. The Malawian trial compares continued CTX with stopping CTX, but is not placebo-controlled; instead weekly chloroquine (CQ) prophylaxis is substituted for CTX. Results are expected in 2015.

## Conclusion

Few studies have investigated the effect of CTX on malaria in patients on ART; these studies show a trend towards a beneficial effect of CTX on malaria. Only 2 of the reviewed studies were randomised and they were the only ones specifically designed to investigate this association. Most of the reviewed studies were subject to bias and confounding, and the clinical relevance of malaria experienced by patients stable on ART who are not on CTX prophylaxis is unclear.
